# Magnetic Mixed Micelles Composed of a Non-Ionic Surfactant and Nitroxide Radicals Containing a d-Glucosamine Unit: Preparation, Stability, and Biomedical Application

**DOI:** 10.3390/pharmaceutics11010042

**Published:** 2019-01-19

**Authors:** Kota Nagura, Yusa Takemoto, Fumi Yoshino, Alexey Bogdanov, Natalia Chumakova, Andrey Kh. Vorobiev, Hirohiko Imai, Tetsuya Matsuda, Satoshi Shimono, Tatsuhisa Kato, Naoki Komatsu, Rui Tamura

**Affiliations:** 1 Graduate School of Human and Environmental Studies, Kyoto University, Kyoto 606-8501, Japan; k.nagura1005@gmail.com (K.N.); vahadrr@gmail.com (Y.T.); shimono.satoshi.4r@kyoto-u.ac.jp (S.S.); kato.tatsuhisa.6e@kyoto-u.ac.jp (T.K.); 2 Department of Obstetrics and Gynecology, Shiga University of Medical Science, Shiga 520-2192, Japan; iwamatsu@belle.shiga-med.ac.jp; 3 Department of Chemistry, M.V. Lomonosov Moscow State University, Moscow 119991, Russian Federation; avbgdn@gmail.com (A.B.); harmonic2011@yandex.ru (N.C.); a.kh.vorobiev@gmail.com (A.K.V.); 4 Graduate School of Informatics, Kyoto University, Kyoto 606-8501, Japan; imai@sys.i.kyoto-u.ac.jp (H.I.); tetsu@i.kyoto-u.ac.jp (T.M.)

**Keywords:** nitroxide radical, magnetic resonance imaging, glucosamine, cancer, micelle

## Abstract

Metal-free magnetic mixed micelles (mean diameter: < 20 nm) were prepared by mixing the biocompatible non-ionic surfactant Tween 80 and the non-toxic, hydrophobic pyrrolidine-*N*-oxyl radicals bearing a d-glucosamine unit in pH 7.4 phosphate-buffered saline (PBS). The time-course stability and in vitro magnetic resonance imaging (MRI) contrast ability of the mixed micelles was found to depend on the length of the alkyl chain in the nitroxide radicals. It was also confirmed that the mixed micelles exhibited no toxicity in vivo and in vitro and high stability in the presence of a large excess of ascorbic acid. The in vivo MRI experiment revealed that one of these mixed micelles showed much higher contrast enhancement in the proton longitudinal relaxation time (*T*_1_) weighted images than other magnetic mixed micelles that we have reported previously. Thus, the magnetic mixed micelles presented here are expected to serve as a promising contrast agent for theranostic nanomedicines, such as MRI-visible targeted drug delivery carriers.

## 1. Introduction

Non-invasive imaging of living tissue is of great importance in the medical field. The magnetic resonance imaging (MRI) method is one of the most frequently used and important imaging techniques in clinical medicine. In fact, the use of MRI contrast agents plays a crucial role in accurately evaluating physiological and pathological changes. The majority of MRI contrast agents approved by the US Food and Drug Administration (FDA) are gadolinium-based contrast agents (GBCAs) such as Magnevist (a Gd^III^ complex agent) [1,2,3]. Although they are used on a daily basis, this modality still faces many challenges [4,5,6,7,8]. For example, people with moderate to advanced kidney failure are in danger of developing nephrogenic systemic fibrosis through the use of GBCAs. Thus, it is urgently required to exploit novel agents that exhibit adequate contrast enhancement with a very low risk.

**Figure 1:**
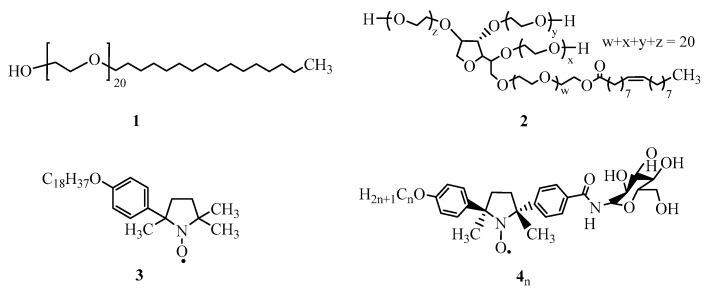
Molecular structures of non-ionic surfactants Brij 58 (**1**) and Tween 80 (**2**), and nitroxide radicals **3** and **4**_n_ (*n* = 14, 16, and 18). Compounds **4**_n_ are a ca. 1:1 mixture of d–(*R*,*R*) and d–(*S*,*S*) diastereomers, see the Supporting Information for the synthesis and characterization.

Metal-free magnetic nanoparticles containing nitroxide radicals as a spin source have attracted great interest since the 1980s [9] because of their lack of toxicity, despite the imaging ability being less compared to Gd^III^ complex agents [10] and their having less reduction resistance to antioxidants such as ascorbic acid and glutathione [11]. However, the reduction resistance should potentially improve through the molecular design and/or the micelle construction of nitroxide radicals [12,13,14,15,16,17]. In this context, we have recently prepared metal-free magnetic mixed micelles comprised of a surfactant, Brij 58 (**1**) or Tweens 80 (**2**), and pyrrolidine-*N*-oxyl radical **3**, namely **1**/**3** or **2**/**3** (Figure 1), according to a simple experimental procedure [18,19]. These micelles showed high colloidal stability, reduction resistance to ascorbic acid, and contrast enhancement in the *T*_1_-weighted MRI in phosphate-buffered saline (PBS) in vitro and in vivo. The mixed micelle **2**/**3** was found to be much less toxic than **1**/**3**. Furthermore, additional hydrophobic fluorophores or drugs were stably encapsulated inside the mixed micelles. Although passive targeting can be expected due to the micelle size (10–20 nm), the micelles that we prepared did not possess any active targeting site for tumor.

Herein, we report on the novel metal-free mixed micelles including nitroxide radicals **4**_n_ conjugated with a d-glucosamine unit as a tumor targeting site, because d-glucosamine derivatives are well-known to accumulate in tumor cells [20,21,22,23]. The obtained magnetic mixed micelles showed little toxicity, excellent in vitro MRI contrast ability, and high stability in the presence of an excess amount of ascorbic acid. When applied to in vivo imaging for healthy mice, bright MRI contrast enhancement was observed in the liver.

## 2. Results and Discussion

### 2.1. Preparation, Stability and In Vitro MRI Contrast Ability of ***2**/**4**_n_*

The nitroxide radicals **4**_n_ (*n* = 14, 16, and 18 in Figure 1, Figures S1 and S2) were synthesized by condensation of the racemic benzoic acid derivatives of the nitroxide radicals (a 1:1 mixture of (*R*,*R*) and (*S*,*S*) enantiomers) [24,25] and d-tetraacetylglucosamine [26,27,28], followed by deacetylation (Schemes S1 and S2 in the Supplementary Information).

**Figure 2:**
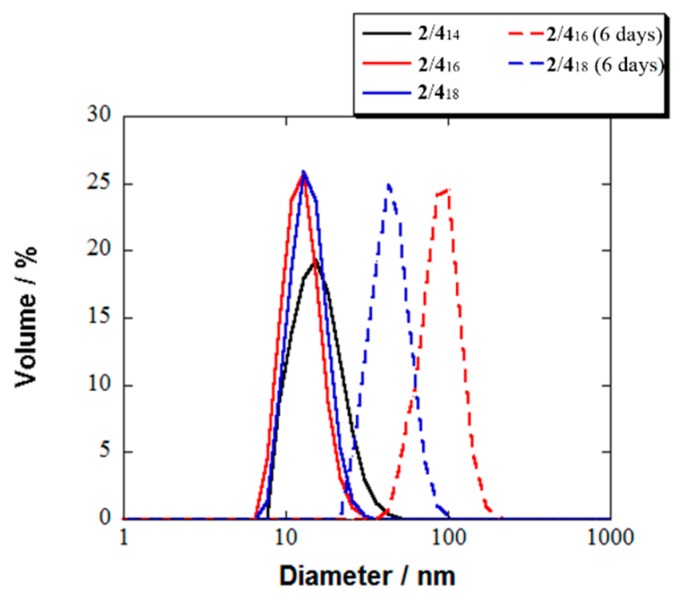
Mean diameters of mixed micelles **2**/**4**_n_ (*n* = 14, 16, and 18) determined by DLS at 25 °C in PBS (black solid line: **2**/**4**_14_ just after preparation, red solid line: **2**/**4**_16_ just after preparation, blue solid line: **2**/**4**_18_ just after preparation, red dashed line: **2**/**4**_16_ after 6 days, blue dashed line: **2**/**4**_18_ after 6 days). See the Supplementary Information for experimental details.

The mixed micelles **2**/**4**_n_ (Figure 1) were prepared at a concentration of 10 mM for each component in the PBS according to the procedure described in the Supplementary Information. The stability of the micelles was found to depend on the length of the alkyl chain (*n* = 14, 16, and 18) in the radicals **4**_n_ (Table 1 and Figure 2). The **2**/**4**_16_ and **2**/**4**_18_ were formed as a clear dispersion immediately after preparation and their mean diameter gradually increased up to 92 and 45 nm after one week, respectively (Table 1). The micelle **2**/**4**_14_ collapsed within one day to give white precipitates of **4**_14_ after 24 h. From these results, summarized in Table 1, the relative stability of the micelles **2**/**4**_n_ in PBS was in the following order: **2**/**4**_18_ > **2**/**4**_16_ > **2**/**4**_14_. The similar dependence of the micellar stability on the alkyl chain length in the nitroxide radicals **4**_n_ was also observed in the cases of the mixed micelles **1**/**3** and **2**/**3** [18,19]. The mean diameters of the resulting magnetic mixed micelles **2**/**4**_n_ in PBS were determined to be 13 to 16 nm by DLS analysis (Table 1 and Figure 2). Their mean diameters fell in a range of 10–100 nm, which is required for the most prolonged blood circulation time. 

Importantly, the once-precipitated sample of **2**/**4**_14_ was revived to the original clear dispersion with the same diameter (16 nm) by just heating it with full reproducibility (Table 1). The micelle **2**/**4**_14_ turned out to be easily available as a clear dispersion even after the long-term preservation of the precipitated sample.

**Figure 3:**
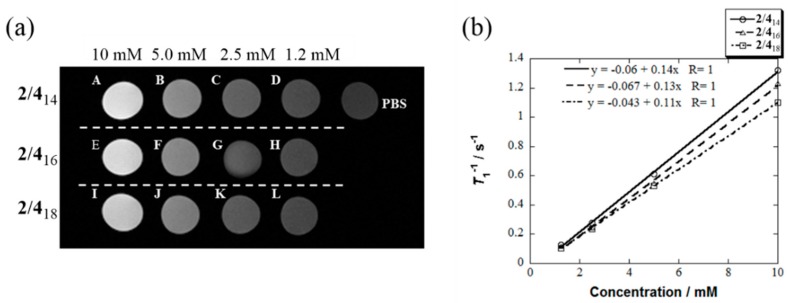
(**a**) (A–D) *T*_1_-weighted MRI phantom images of **2**/**4**_14_ (**4**_14_: 1.2 to 10 mM), (E–H) **2**/**4**_16_ (**4**_16_: 1.2 to 10 mM) and (I–L) **2**/**4**_18_ (**4**_18_: 1.2 to 10 mM) in PBS, and control PBS at 7.0 T and 25 °C. (**b**) Plots of *T*_1_^−1^ vs concentrations of **2**/**4**_14_ (solid line), **2**/**4**_16_ (dashed line) and **2**/**4**_18_ (dashed and dotted line) at 1.2, 2.5, 5.0, 10 mM for each component. The *r*_1_ was determined from the slope of each line. See the Supplementary Information for experimental details.

The dependence of the alkyl chain length in **4**_n_ on the longitudinal relaxivity (*r*_1_) of **2**/**4**_n_ was determined from the relaxation time (*T*_1_) as a function of the concentration at 25 °C by using an MRI machine at 7.0 T. Sufficiently bright *T*_1_-weighted MR phantom images were obtained at a concentration of 10 mM of the magnetic mixed micelles **2**/**4**_14_, **2**/**4**_16_, and **2**/**4**_18_ as compared with that of the control PBS (panel A, E, and I in Figure 3a). This result implies that **2**/**4**_n_ may show a distinct MRI contrast enhancement in vivo in this concentration or higher. The linear regression analysis yielded *r*_1_ = 0.14, 0.13, and 0.11 mM^−1^s^−1^ for **2**/**4**_14_, **2**/**4**_16_ and **2**/**4**_18_, respectively (Figure 3b). That is, the MRI contrast ability of the micelle **2**/**4**_n_ in PBS was in the following order: **2**/**4**_14_ > **2**/**4**_16_ > **2**/**4**_18_. The mixed micelle **2**/**4**_14_ was used for further experiments for the following two reasons: (1) **2**/**4**_14_ exhibited superior in vitro MRI-enhanced ability to those of **2**/**4**_16_ and **2**/**4**_18_, and (2) the clear dispersion was fully revived in a reproducible manner by simply heating the precipitated sample. Although the stability of the **2**/**4**_14_ was less than that of **2**/**4**_16_ and **2**/**4**_18_ as mentioned above (Table 1), we gave priority to the MRI-enhanced ability over the stability.

**Figure 4:**
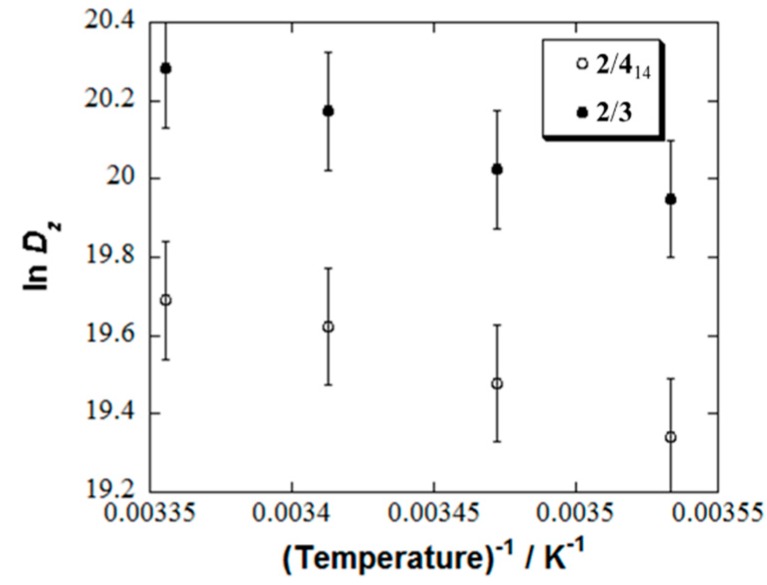
Temperature dependence of rotation diffusion coefficient *D_z_* of **4**_14_ in **2**/**4**_14_ and **3** in **2**/**3**. The data of **2**/**3** was cited from reference 19. See the Supplementary Information for experimental details of **2**/**4**_14_.

These *r*_1_ values were much larger than those of micelles **2**/**3** and **1**/**3** (*r*_1_ = 0.07, and 0.09 mM^−1^s^−1^, respectively, at 7.0 T), which we reported previously, although they were much lower than those of Gd^III^ complex agents [6]. These experimental results could be interpreted in terms of the slower rotation diffusion of **4**_14_ than that of **3** inside the mixed micelles [29,30,31]. In order to compare the rotation diffusion mobility between radicals **4**_14_ and **3** inside the micelles, the electron paramagnetic resonance (EPR) spectra of radical **4**_14_ or **3** in the micelles consisting of a 1:0.01 molar ratio of surfactant **2** and **4**_14_ or **3** were measured in the temperature range 263–298 K and then were numerically simulated as described in the Supplementary Information (Figure 4 and Figure S3, and Table S1). The temperature dependence of the rotation diffusion mobility was successfully described by Arrhenius law with the values of activation energy (*E^a^_z_*) shown in Table 2, indicating that **4**_14_ showed slower rotational diffusion inside the micelle to produce a highly enhanced MRI compared to **3**. The better *r*_1_ of **2**/**4**_14_ than **2**/**4**_16_ and **2**/**4**_18_ mentioned above might be interpreted by the slower rotation diffusion mobility of **4**_14_ than **4**_16_ and **4**_18_.

### 2.2. Reduction Resistivity of ***2**/**4**_14_* in the Presence of Ascorbic Acid

**Figure 5:**
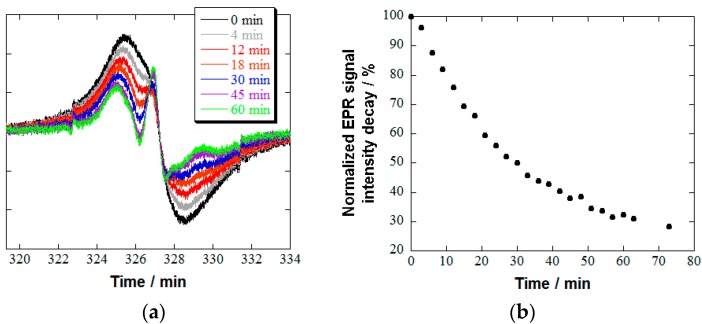
(**a**) Time-course of EPR spectra and (**b**) the reduction resistance of **4**_14_ in **2**/**4**_14_ to a large excess of ascorbic acid (20 equiv based on **4**_14_) in PBS at 25 °C. The normalized signal intensity decay was evaluated by a double-integration method. See the Supplementary Information for experimental details.

The concentration of ascorbic acid in the healthy adult serum was reported to be kept in the range of 14.9–52.8 μM by a daily intake of ascorbic acid (60 mg) [32]. When nitroxide radicals were applied to the in vivo MRI measurement, radical reduction occurred and resulted in a significant decrease in the MRI contrast [33,34,35]. For example, 2,2,6,6-tetramethylpiperidine-*N*-oxyl (TEMPO) derivatives, such as 4-oxo-2,2,6,6-tetramethylpiperidine-*N*-oxyl (TEMPONE) and 4-hydroxy-2,2,6,6-tetramethylpiperidine-*N*-oxyl (TEMPOL), were reduced rapidly (half-life (*τ*_1/2_) < 2 min) to the corresponding hydroxylamines in the presence of ascorbic acid [36]. In our molecular design, we expected that the interplay between four long hydrophilic tails in **2** and four neighboring substituents in **4**_14_ should enhance the reduction resistance to ascorbic acid sterically. The decay of **4**_14_ in **2**/**4**_14_ in response to a large excess of ascorbic acid (20 equiv based on **4**_14_) in PBS was monitored by EPR spectroscopy (Figure 5). As expected, the *τ*_1/2_ of **2/4**_14_ (30 min) was almost comparable to that of **2**/**3** (33 min) and much longer than that of **1**/**3** (7 min) [19]. 

### 2.3. Biomedical Application of ***2**/**4**_14_*

**Figure 6:**
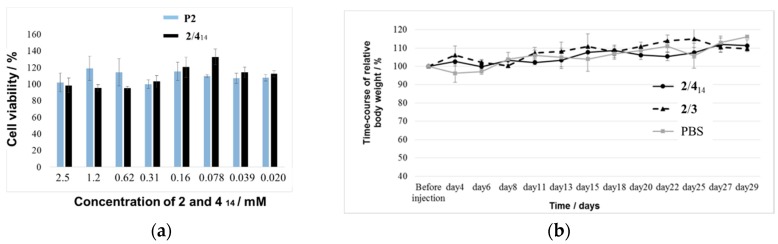
(**a**) In vitro cell viability of **2**/**4**_14_ and **P2** by using the CCK-8 kit after incubation for 24 h at 37 °C under 5% CO_2_ and (**b**) in vivo toxicity of **2**/**4**_14_ for healthy ICR mice weight (three mice for each of **2**/**4**_14_, **2**/**3** and control PBS) as a function of time after injection of 200 μL of mixed micelles (40 mM for each component) in PBS or PBS. See the Supplementary Information for experimental details.

Since biocompatibility is a prerequisite of using the magnetic mixed micelles as an MRI contrast agent, the cancer cell viability of **2**/**4**_14_ was assessed by the CCK-8 assay at the initial concentration of 2.5 mM for **2** and **4**_14_ and compared with those of pure micelle of **2**, designated as **P2**, (Figure 6a). Both **P2** and **2**/**4**_14_ exhibited little cytotoxicity to HeLa cells at concentrations up to 2.5 mM, demonstrating that **2**/**4**_14_ is an appropriate candidate for in vivo experiment. In addition, the body weights gradually increased in the healthy Institute of Cancer Research (ICR) mice over one month after injection of **2**/**4**_14_, **2**/**3** and PBS (Figure 6b). It was concluded that mixed micelles **2**/**4**_14_ can serve as a bio-compatible MRI contrast agent similar to **2**/**3**. 

**Figure 7:**
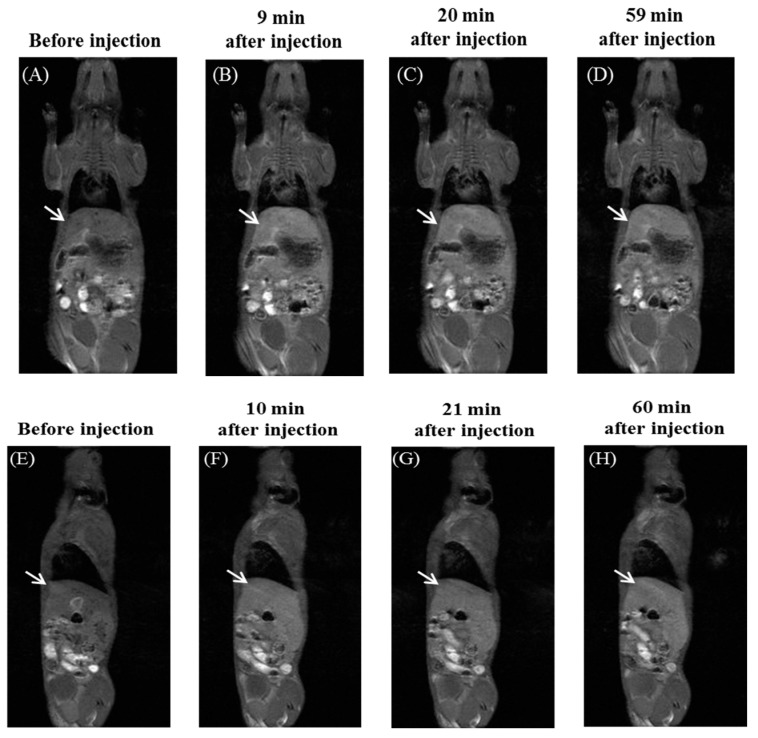
Time-course of coronal (panels **A**–**D**) and sagittal (panels **E**–**H**) *T*_1_-weighted MR images of an ICR mouse before and after injection of 200 μL of **2**/**4**_14_ (40 mM) in PBS. Distinct contrast enhancement was observed in the liver of the mouse (indicated by white arrows). See the Supplementary Information for experimental details.

Finally, the in vivo MRI experiment using **2**/**4**_14_ was performed for healthy ICR mice. Bright MRI contrast enhancement was observed in the liver in both coronal and sagittal planes over 1 h with high reproducibility (Figure 7). This result reveals that the magnetic mixed micelle **2**/**4**_14_ is effective as an in vivo *T*_1_-weighted MRI contrast agent. The prolonged MRI enhancement observed for **2**/**4**_14_ is attributed to the high resistance to reducing agents as described above.

## 3. Conclusions

We prepared highly robust and biocompatible metal-free magnetic mixed micelles which are composed of non-ionic surfactant **2** and hydrophobic nitroxide radical **4**_n_ in PBS. The time-course stability and in vitro MRI contrast ability of the mixed micelles was found to depend on the length (*n*) of the alkyl chain in the nitroxide radicals. In addition, the mixed micelle **2**/**4**_14_ showed a considerable reduction resistance to a large excess of ascorbic acid, little toxicity, and sufficient contrast enhancement in the *T*_1_-weighted MRI in vivo. Such highly biocompatible magnetic mixed micelles composed of nitroxide radicals bearing a d-glucosamine unit are expected to be utilized as a low-molecular-weight cancer targeted MRI contrast agent in line with the theranostic applications of micelles, which have recently been attracting increasing interest [37,38,39].

## Figures and Tables

**Table 1:** Mean diameters and colloidal stability of the mixed micelles **2**/**4**_n_ (*n* = 14, 16, and 18) in phosphate-buffered saline (PBS) at 30 °C.

Micelle	2/4_14_	2/4_16_	2/4_18_
Diameter by DLS	16 nm ^a^	13 nm ^a^ 92 nm ^c^	14 nm ^a^ 45 nm ^c^
Colloidal stability	Dispersion ^a^ 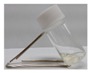	Dispersion ^a^ 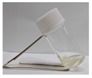	Dispersion ^a^ 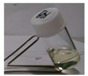
	Precipitates ^b^ 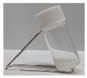		
	Dispersion ^d^ 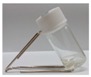	Dispersion ^c^ 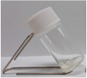	Dispersion ^c^ 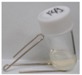

^a^ Immediately after preparation. ^b^ After 24 h of preparation. ^c^ After 6 days of preparation. ^d^ After heating the precipitates.

**Table 2:** The effective activation energy of the rotation diffusion of **4**_14_ in **2**/**4**_14_ and **3** in **2**/**3**.

Mixed Micelle	*E^a^_z_*/kJmol^−1^
**2**/**4**_14_	21.1 ± 1.0
**2**/**3**^a^	18.4 ± 0.4

^a^ Previously reported value [19].

## Supplementary Materials

The following are available online at http://www.mdpi.com/1999-4923/11/1/42/s1, Scheme S1: Synthesis of **7**_n_ (*n* = 14, 16, and 18); Scheme S2: Synthesis of **4**_n_ (*n* = 14, 16, and 18); Figure S1: FT-IR spectra (KBr) of (**a**) **4**_14_ (**b**) **4**_16_ and (**c**) **4**_18_; Figure S2: HPLC charts of (**a**) **4**_14_, (**b**) **4**_16_, and (**c**) **4**_18_; Figure S3: Representative EPR spectra of **2**/**4**_14_ (a molar ratio of 1:0.01) and the results of their computer simulation at high temperatures; Table S1: Rotation diffusion coefficients and the angles determining the position of the main rotation axis in *g*-tensor frame for radical **4**_14_ in **2**/**4**_14_ (a molar ratio of 1:0.01).
