# Supplementary material for: Magnetic Mixed Micelles Composed of a Non-Ionic Surfactant and Nitroxide Radicals Containing a d-Glucosamine Unit: Preparation, Stability, and Biomedical Application
Source: Pharmaceutics. 2019 Jan 19;11(1):42. doi: 10.3390/pharmaceutics11010042 (PMC6359449; doi:10.3390/pharmaceutics11010042)
Supplement: Supplementary file 1 [file pharmaceutics-11-00042-s001.pdf]

pharmaceutics

MDPI

Supplementary Materials: Magnetic Mixed Micelles Composed of a Non-Ionic Surfactant and Nitroxide Radicals Containing a D-Glucosamine Unit: Preparation, Stability, and Biomedical Application

1. General

The surfactant **2** (Tween 80 (DKS, Kyoto, Japan)) was used as received. Unless otherwise noted, solvents and reagents were reagent grade and used without further purification. Tetrahydrofuran (THF) (Kanto Chemical, Tokyo, Japan) used for EPR spectroscopy or Grignard reactions was distilled from sodium/benzophenone ketyl under argon. EPR spectra were recorded on JEOL JES-RE2X (JEOL, Tokyo, Japan) and Bruker EMX Plus (Bruker Biospin, Rheinstetten, Germany). FT-IR spectrometry was performed with a Shimadzu IRSpirit instrument (Shimadzu, Kyoto, Japan) using a dry KBr powder. Elementary analysis was performed with a Yanaco CHN Corder MT-6 instrument (Yanaco, Kyoto, Japan) at the Center of Organic Elemental Microanalysis of Kyoto University.

2. Synthesis

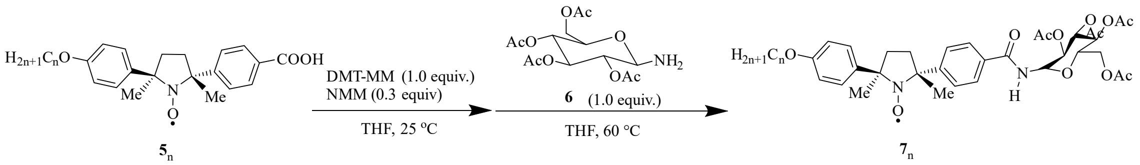

Scheme S1. Synthesis of **7**n (n = 14, 16, and 18).

As a typical example, $\mathbf{7}_{18}$ was obtained as a ca. 1:1 mixture of D-(R,R) and D-(S,S) diastereomers as follows: 4-(4,6-Dimethoxy-1,3,5-triazin-2-yl)-4-methylmorpholinium chloride (DMT-MM) (174 mg, 0.629 mmol) (TCI, Tokyo, Japan) was added to a solution of (±)-$\mathbf{5}_{18}$ (330 mg, 0.572 mmol) [1] and N-methylmorpholine (NMM) (17.4 mg, 0.172 mmol) (TCI, Tokyo, Japan) in THF at 25 °C. After stirring for 15 min, a solution of D-tetraacetylglucosamine (**6**) (238 mg, 0.686 mmol, 1.2 equiv) [2,3,4] was added, and the mixture was stirred for 16 h at 70 °C. The resultant mixture was extracted three times with CH_2Cl_2 (15 ml × 3). The combined organic layer was dried over $MgSO_4$ and filtered, and the solvent was evaporated under reduced pressure. The residual solid was purified by flash column chromatography on silica gel eluting with $CHCl_3$/hexane/diethyl ether (50:40:10 to 60:20:20, v/v/v) to give $\mathbf{7}_{18}$ as a yellow solid (175 mg, 0.193 mmol, 34%).

Similarly, $\mathbf{7}_{14}$ and $\mathbf{7}_{16}$ were prepared in 52% and 74% yields, respectively, as yellow solids.

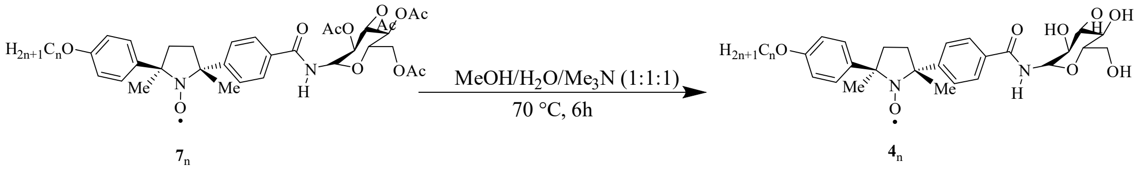

Scheme S2. Synthesis of **4**n (n = 14, 16, and 18).

Subsequently, $\mathbf{7}_{18}$ (175 mg, 0.193 mmol) was dissolved in a mixture of H_2O, MeOH and trimethylamine (2 mL each) at 25 °C and the mixture was stirred overnight at 25 °C. The resultant solution was acidified with 5 w% H_2SO_4 to pH 6–7 and extracted three times with CH_2Cl_2 (15 ml × 3). The combined organic layer was dried over $MgSO_4$ and filtered, and the solvent was evaporated under reduced pressure. The residual solid was purified by flash column chromatography on silica

gel eluting with $CHCl_3$/MeOH (90:10 v/v) to give **4**$_{18}$ as a yellow solid (84 mg, 0.114 mmol, 59%). Similarly, **4**$_{14}$ and **4**$_{16}$ were prepared in 41% and 54% yields, respectively, as yellow solids. The existence of functional groups in **4**$_n$ (*n* =14, 16, and 18) was confirmed by FT-IR spectroscopy (Figure S1). The HPLC analyses of products **4**$_n$ (*n* = 14, 16, and 18) verified the high purity of each diasteromer mixture (Figure S2).

4$_{14}$: EPR (1.0×10^{-4} M in THF, 25 °C) g = 2.0063, a_N = 1.32 mT. Anal. Calcd for $C_{39}H_{59}N_2O_8$ (%): C, 68.49; H, 8.70; N, 4.10. Found (%): C, 68.15; H, 8.97; N, 3.66.

4$_{16}$: EPR (1.0×10^{-4} M in THF, 25 °C) g = 2.0065, a_N = 1.32 mT. Anal. Calcd for $C_{41}H_{63}N_2O_8\bullet H_2O$ (%): C, 67.46; H, 8.98; N, 3.84. Found (%): C, 67.19; H, 9.07; N, 3.71.

4$_{18}$: EPR (1.0×10^{-4} M in THF, 25 °C) g = 2.0065, a_N = 1.32 mT. Anal. Calcd for $C_{43}H_{67}N_2O_8$ (%): C, 69.79; H, 9.13; N, 3.79. Found (%): C, 69.60; H, 9.39; N, 3.53.

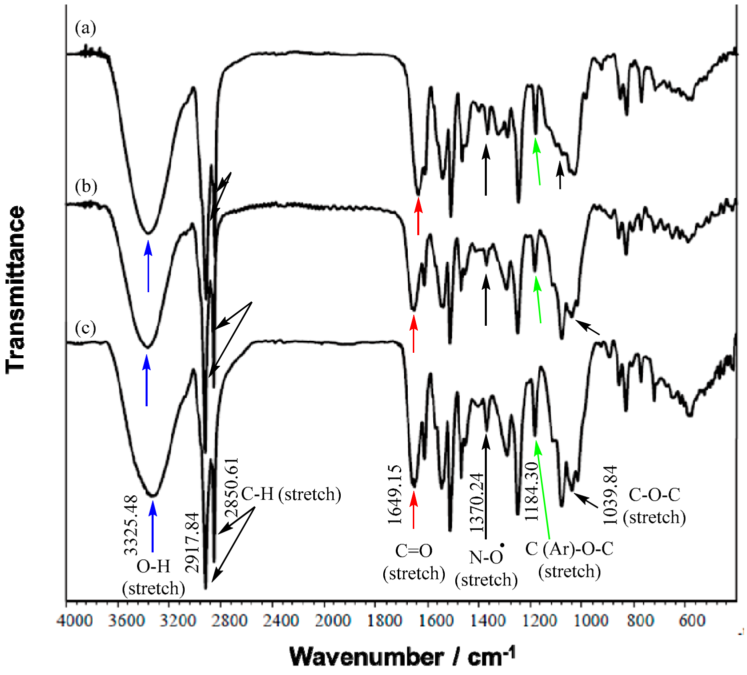

Figure S1. FT-IR spectra (KBr) of (**a**) **4**$_{14}$ (**b**) **4**$_{16}$ and (**c**) **4**$_{18}$.

(a)

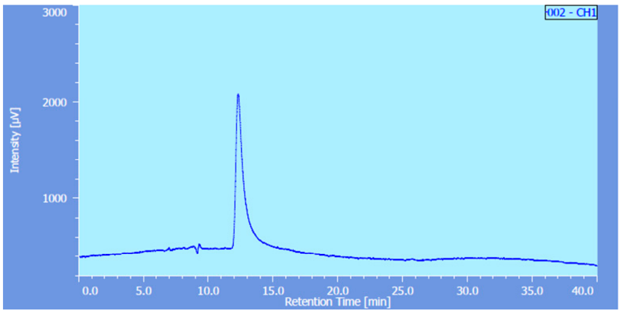

(b)

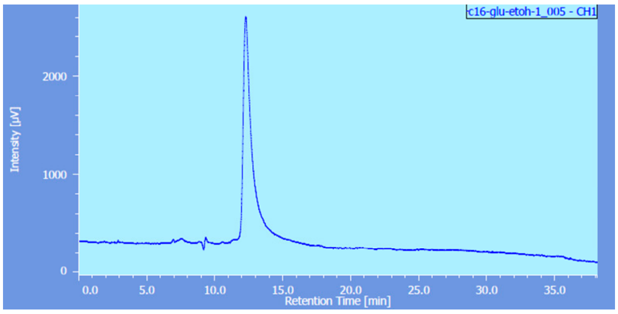

(c)

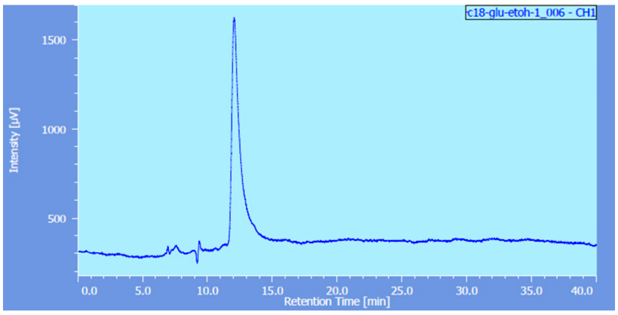

Figure S2. HPLC charts of (**a**) **4**$_{14}$, (**b**) **4**$_{16}$, and (**c**) **4**$_{18}$. HPLC analysis was carried out using a silica gel column (YMC-Pack SIL-06, 0.46 x 30 cm) (YMC, Kyoto, Japan) at 30 °C, a mixture of hexane and ethanol (6:4 v/v) as the mobile phase at a flow rate of 0.5 mL/min, and UV-vis spectrometer (254 nm) as the detector.

3. Preparation of Magnetic Mixed Micelles 2/4$_n$ (n = 14, 16, and 18) in PBS

As a typical example, **2/4**$_{14}$ (10 mM for each component) was prepared in PBS (FUJIFILM Wako Pure Chemical, Osaka, Japan) as follows. To a glass vial containing **4**$_{14}$ (13.67 mg, 20 µmol) was added **3** (10 mM) dissolved in PBS (2.0 mL). The mixture was subjected to sonication (Branson Model 5800, frequency 40 kHz) (Branson, Danbury, USA) for more than 10 min at 25 °C to give a white suspension. Then the suspension was heated for more than 10 min at 90 °C using a water bath to form a clear yellow dispersion of **2/4**$_{14}$, which was passed through a 0.45 µm membrane filter. **2/4**$_{16}$ and **2/4**$_{18}$ (10 mM each), and **2/4**$_{14}$ (40 mM each) in PBS were prepared by the same procedure.

4. DLS Measurement (Table 1 and Figure 2)

The mean diameter of mixed micelles was determined using a UPA-UT151 instrument (MicrotrackBEL, Osaka, Japan) at 25 °C. After the samples were passed through a 0.45 µm ϕ disposable membrane filter, the particle size was measured in PBS using an attached cuvette. The mean diameter was calculated on volume and number weighted averages from five measurements for each sample. Only the volume average size distributions are described in this paper. The polydispersity index was not available using the present instrument.

5. EPR Spectroscopy

5.1. Determination of the rotation diffusion coefficient (D_z) (Figure 4)

The EPR spectra of **2/4**$_{14}$ (a ratio of 1:0.01 of **2–4**$_{14}$) were recorded at 100 K and in the temperature range 263–298 K on a Bruker EMX Plus spectrometer (X-band). The variable temperature unit (Bruker) was used to control temperature. Microwave powers were chosen carefully, especially in the low-temperature measurement, to prevent the signal saturation arising from the prolonged spin-lattice relaxation time of a radical magnetization vector. Modulation amplitudes were kept below 1/2 to 1/3 times of peak-to-peak line widths. For the experiment at 298 K, the samples were placed into glass capillary tubes (~1 mm ϕ). For the experiment at 100 K, the samples in the plastic tubes (~ 4 mm ϕ) were dipped quickly in liq. N_2 to be frozen. The rotation diffusion coefficients and angles (beta and gamma) that define the position of the rotation axis of the probe in the g-tensor frame were determined by nonlinear-least squares simulation of the experimental ESR spectra, i.e., by minimizing the discrepancy (sum of squared deviations) between the experimental and the simulated spectra. The errors of determined parameters were estimated based on the covariance matrix at the point of minimum. The magnetic parameters of the radicals (principal values of *g*- and hfc-tensors) were determined independently from the spectra recorded at 100 K. The representative result of the simulation of several high-temperature spectra is presented in Figure S3. The detailed EPR fitting procedure was described in our previous paper [5].

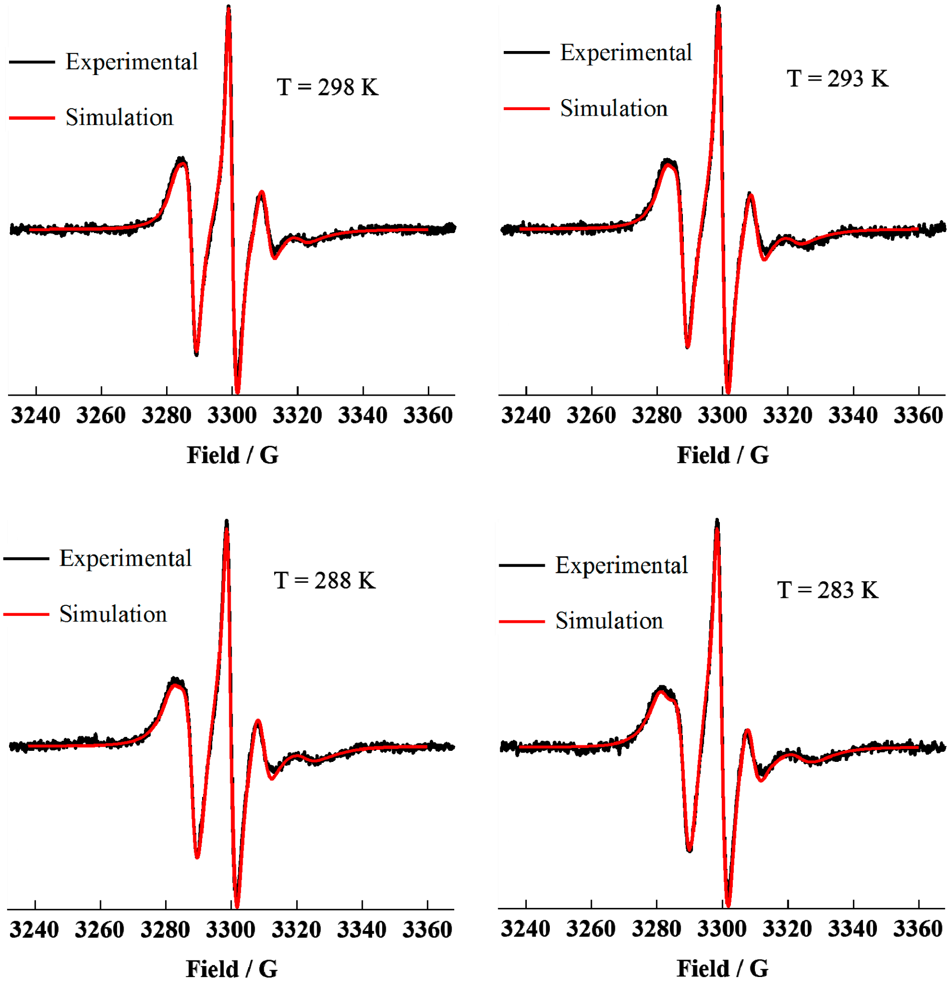

Figure S3. Representative EPR spectra of **2/4**$_{14}$ (a molar ratio of 1:0.01) and the results of their computer simulation at high temperatures. Black lines denote the experimental spectra, red lines represent the results of the simulation.

Table S1. Rotation diffusion coefficients and the angles determining the position of the main rotation axis in *g*-tensor frame for radical $\mathbf{4}_{14}$ in $\mathbf{2}/\mathbf{4}_{14}$ (a molar ratio of 1:0.01).

K	D_z, 10^8 s^{-1}	*Beta* [a], (±2°) [b]	*Gamma* [a], (±2°) [b]	*Discrepancy* (×10^{-7})
298	3.6 ± 0.5 [b]	45	75	0.741
293	3.3 ± 0.5	45	73	0.363
288	2.9 ± 0.4	44	90	0.333
283	2.5 ± 0.4	43	90	0.414
281	2.3 ± 0.3	43	90	0.202
279	2.2 ± 0.3	42	90	0.409
277	2.1 ± 0.3	41	90	0.170
275	1.8 ± 0.3	42	90	0.157
273	1.8 ± 0.3	41	90	0.158
271	1.9 ± 0.3	38	90	0.213
269	1.4 ± 0.2	40	90	0.110
267	1.4 ± 0.2	39	90	0.165
265	1.2 ± 0.2	37	90	0.249
263	1.2 ± 0.2	36	90	0.115

[a] Euler angles describe the transformation of rotation frame to *g*-tensor frame [6,7]. [b] Error values are presented.

5.2. Measurement of the Reduction Resistance of the Mixed Micelles to a Large Excess of Ascorbic Acid (Figure5)

To $\mathbf{2}/\mathbf{4}_{14}$ (10 mM each) in PBS (1.5 mL) was added 20 equiv of ascorbic acid. The mixture was transferred to a capillary tube (1 mm *ϕ*) immediately, followed by the measurement of EPR spectra during the process of the radical reduction. The spectra were collected with the following parameters; 1.00 mW of microwave power, 0.079 mT of modulation, 0.10 s of time constant, and 120 sec of sweep time. This experiment was performed twice and the high reproducibility was confirmed.

6. Evaluation Method of *In Vitro* Cytotoxicity (Figure 6a)

HeLa cells (8,200 cells in 100 µL per well) were seeded in a 96-well tissue culture plate with Dulbecco's modified Eagle's medium (DMEM) (FUJIFILM Wako Pure Chemical, Osaka, Japan) containing 10% fetal bovine serum (FUJIFILM Wako Pure Chemical, Osaka, Japan) and 1% penicillin/streptomycin (FUJIFILM Wako Pure Chemical, Osaka, Japan) and grown for 24 h at 37 °C with 5% CO_2. Then, each well was treated with a PBS dispersion (10 µL) of **P2** or $\mathbf{2}/\mathbf{4}_{14}$. The initial dispersion of the mixed micelles containing 2.5 mM of **2** and $\mathbf{4}_{14}$ and diluted dispersion of 1.2, 0.62, 0.31, 0.16, 0.078, 0.039, and 0.020 mM were applied to the cells before incubation. After incubation for 24 h at 37 °C under 5% CO_2, the compounds shown above were removed and then cell toxicity was assayed using CCK-8 kit according to the manual provided by the kit manufacturer (Dojindo Molecular Technologies, Kumamoto, Japan).

7. *In Vivo* Toxicology Test (Figure 6b)

The protocols for the animal experiments were approved by the Shiga University of Medical Science (approval number: 2018-4-1) and were carried out in accordance with the National Institutes of Health Animal Care and Use Protocol (NIH, Bethesda, MD, USA). Female ICR mice (aged 7 weeks, body weight 32–34 g) were supplied from Japan SLC Inc. (Shizuoka, Japan) and nine mice were used for the toxicological experiments. Three mice were housed per cage in climate-controlled, circadian-rhythm-adjusted rooms, and were allowed access to food and water ad libitum.

To evaluate the toxicity of the mixed micelles to an animal, 200 µL of $\mathbf{2}/\mathbf{4}_{14}$ (40 mM each), or controls **2**/**3** (40 mM) in PBS or PBS were intravenously administered to nine mice which were separated into three groups under anesthesia with 1.5% isoflurane. The body weight of each mouse

(30–32 g) before injection was normalized to 100 in order to make valid comparisons, and the body weight changes of three different ICR mice were monitored for **2**/**4**$_{14}$, **2**/**3** (employed as *in vivo* MRI contrast agent in our previous report [5]) and PBS over one month. The mean body weight values of three measurements each day were plotted with the standard deviation represented by error bars.

8. Evaluation Method of the *In Vitro* MRI Experiment (Figure 3)

In vitro MRI measurement was conducted on a 7-Tesla preclinical scanner (BioSpec 70/20 USR; Bruker BioSpin MRI GmbH, Ettlingen, Germany). The initial PBS dispersion of **2**/**4**$_n$ (10 mM each) and diluted dispersions of 5.0, 2.5, and 1.2 mM were prepared. These mixed micelles were transferred to plastic tubes and fixed in a sample holder. The MR relaxometry was conducted at 25 °C. MR phantom images were obtained by a rapid acquisition with relaxation enhancement (RARE) pulse sequence with variable repetition time (TR) (echo time (TE) = 11 ms, rare factor = 2, repetition time (TR) = 5,000, 3,000, 1,500, 800, 400, 200, 100, and 50 ms, field of view = 80 × 40 mm^2, acquisition matrix size = 256 × 128, and slice thickness = 2.00 mm). In the acquired images, the region-of-interest (ROI) was set on each tube and the mean signal intensities within the ROI were measured for the TR-varied images. T_1 values were calculated by fitting a saturation recovery equation to the plot of signal intensity versus TR using the image sequence analysis tool in ParaVision 5.1 (Bruker BioSpin).

9. *In Vivo* MRI Experiment (Figure 7)

All animal procedures for *in vivo* MRI measurement were conducted in accordance with the guidelines of animal experiments of Kyoto University (approval number: 30−A−9). Male ICR mice (n = 2, aged 7 weeks, body weight 32–34 g; JAPAN SLC. Inc., Shizuoka, Japan) were used. After the induction of anesthesia with isoflurane, mice were placed in a cradle in a prone position. The anesthesia was kept with an inhalation of 2% isoflurane in air at 1.4 L/min through a face mask. Respiratory rate and rectal temperature were continuously measured using a pressure-sensitive respiration sensor and thermistor temperature probe, respectively, and monitored using a monitoring system (Model 1025, MR-compatible Small Animal Monitoring and Gating System; SA Instruments, Inc., NY, USA) with a dedicated software (PC-SAM V.5.12; SA Instruments). The body temperature was maintained by a flow of warm air using a heater system (MR-compatible Small Animal Heating System, SA Instruments).

MRI measurements were conducted on a 7-Tesla preclinical scanner (BioSpec 70/20 USR; Bruker BioSpin MRI GmbH, Ettlingen, Germany). A quadrature transmit-receive volume coil (inner diameter 72 mm, T9562; Bruker BioSpin) was used for MR signal detection. After 200 μL of **2**/**4**$_{14}$ (40 mM each) in PBS were intravenously injected into an anesthetized male ICR mouse (body weight: 32–34 g) lying prone in an animal holder, MRI data were acquired with a dedicated operation software (ParaVision 5.1; Bruker BioSpin). A two-dimensional multi-slice spin-echo pulse sequence with fat suppression was used for acquiring the T_1-weighted images. The acquisition parameters were as follows: repetition time (TR) = 200 ms, echo time (TE) = 6.2 ms, field of view (FOV) = 80 × 40 mm^2, acquisition matrix size = 256 × 128, spatial resolution of 312 μm, coronal and sagittal orientations, slice thickness = 2 mm, number of slices = 8, number of averages = 2, and scan time = 51.2 s.

References

[1]. Uchida, Y.; Uematsu, T.; Nakayama, Y.; Takahashi, H.; Tsue, H.; Tanaka, K.; Tamura, R. Partial resolution of racemic trans-4-[5-(4-alkoxyphenyl)-2,5-dimethylpyrrolidine-1-oxyl-2-yl]benzoic acids by the diastereomer method with (R)- or (S)-1-phenylethylamine. *Chirality* **2008**, *20*, 282–287.

[2]. Zhang, Q.; Lebl, T.; Kulczynska, A.; Botting, N.P. The synthesis of novel hexa-^{13}C-labelled glucosinolates

from [$^{13}C_6$]-D-glucose. *Tetrahedron* **2009**, *65*, 4781–4876.

[3]. Soli, E.D.; Manoso, A.S.; Patterson, M.C.; DeShong, P. Azide and cyanide displacements via hypervalent silicate intermediates. *J. Org. Chem*. **1999**, *64*, 3171–3177.

[4]. Muhizi, T.; Grelier, S.; Coma, V. Synthesis and antibacterial activity of aminodeoxyglucose derivatives against *Listeria innocua* and *Salmonella typhimurium*. *J. Agric. Food Chem*., **2009**, *57*, 8770–8775.

[5]. Nagura, K.; Bogdanov, A.; Chumakova, N.; Vorobiev, A. K.; Moronaga, S.; Imai, H.; Matsuda, T.; Noda, Y.; Maeda, T.; Koizumi, S.; Sakamoto, K.; Amano, T.; Yoshino, F.; Kato, T.; Komatsu, N.; Tamura, R.; Size-tunable MRI-visible nitroxide-based magnetic mixed micelles: Preparation, stability and theranostic application. Submitted for publication.

[6]. Vorobiev, A.K.; Chumakova, N.A. In *Nitroxides - Theory, Experiment and Applications*, Kokorin, A. I., Ed.; INTECH, Rijeka, Croatia, 2012; Chapter 3.

[7]. Budil, D.E.; Lee, S.; Saxena, S.; Freed, J. H. Nonlinear-least-squares analysis of slow-motion EPR spectra in one and two dimensions using a modified levenberg-marquardt algorithm. *J. Magn. Reson., Ser A.* **1996**, *120*. 155–189.
